# Bereaved mothers' experience of expressing and donating breast milk: An interpretative phenomenological study

**DOI:** 10.1111/mcn.13473

**Published:** 2023-03-16

**Authors:** Gráinne Ward, Pauline Adair, Nicola Doherty, David McCormack

**Affiliations:** ^1^ Department of Clinical Psychology Queen's University Belfast Northern Ireland UK

**Keywords:** breast milk, milk banking, neonatal mortality, parent, postpartum, qualitative methods

## Abstract

Perinatal loss is a devastating event for any mother. What is often overlooked is a mothers continued ability to lactate following the death of her child. Donor breast milk is a commodity highly sought after given its value for feeding sick babies resident in Neonatal Intensive Care Units. This study aimed to explore the lived experience of mothers who have expressed and donated their breast milk following the loss of their infant. Seven bereaved mothers who donated milk to the Human Milk Bank in Northern Ireland were recruited. These women took part in semistructured interviews, which explored their experiences of perinatal loss and the role that expression/donation played for them in their grief. Their accounts were analysed using a qualitative interpretative phenomenological analysis (IPA) method. After transcription and analysis, three superordinate themes emerged; (1) fulfilling the mother role; (2) the power of being able to ‘Do’; (3) making good from the bad. The stories of these women reflect the independent and individual nature of grief. Each mother gained a great deal of comfort in having the ability to express milk. For some this created a physical connection to their child, for others, it created time alone to process what had happened and for all, it created a sense of autonomy and ownership in what was otherwise a very turbulent time in their lives.

## Key points

- Bereaved mothers can continue to fulfil their role as a mother and benefit psychologically following perinatal loss by expressing and donating breast milk for the health benefit of other babies.
- Mothers can face barriers to exploring options for donating breast milk following perinatal loss.
- Perinatal and maternity services should develop compassionate and sensitive approaches to support bereaved mothers in their grief including providing relevant information and support to express and donate breast milk.
- Increased awareness of the possible benefits of expression must be disseminated across staff teams to ensure professionals are armed with the knowledge they need to support mothers appropriately.

## 1 INTRODUCTION

### 1.1 Perinatal loss

Death of a child is a devastating loss for any mother, particularly in the case of a stillbirth or neonatal death. This leads to a unique experience of grief as the mother grieves both the loss of her baby and the loss of the maternal role that she had anticipated fulfilling (Cacciatore, 2013). Characteristic features of maternal grief include anger, anxiety, guilt and depression (Shear, 2015). This can often be overwhelming for the mother and has been known to produce long‐lasting effects such as posttraumatic stress symptoms, low mood and sleep disturbance if complications arise during the grief process (Pohlkamp et al., 2019). Complicated grief can be seen in 20%–30% of mothers following perinatal loss (Morris et al., 2019); this happens when a mother fails to integrate the loss of her child and acute grief is prolonged. This preoccupied state can lead to mothers failing to engage in ongoing life (Davidson & Stahls, 2010).

Perinatal loss is often followed by grief reactions from those outside the immediate family, some of which can intensify reactions to a loss, for example, silence, communication difficulties and awkwardness (K. E. Carroll et al., 2014). Experiencing grief is an essential aspect of the healing process; mothers are encouraged by professionals to stay connected to their emotions and actively engage with their grief to find recovery. Uren and Wastell (2002) reported that a mother's capacity to find meaning following a bereavement predicted her ability to move through the grieving process and heal. In some mothers, perinatal loss has been found to elicit positive posttraumatic growth and change, this occurs when a mothers’ fundamental assumptions about life are challenged and adapted (Currier et al., 2012; Tian & Solomon, 2020).

### 1.2 Breast milk and its purpose

A breastfed infant receives numerous benefits regarding infant nutrition, gastrointestinal function, psychological well‐being and immune development (Binns et al., 2016). Breast milk contains ingredients which support immune function and posits antioxidative properties (Martin et al., 2016). Breast milk has all the necessary protective components and nutrients for healthy development in babies alongside the ability to decrease risk of infectious diseases (Ballard & Morrow, 2013).

Breast milk has been positively associated with both short‐ and long‐term outcomes in the neurodevelopment of vulnerable, sick or preterm infants (Ottolini et al., 2020). The World Health Organization (2015) recommends that breastfeeding is the best nutrition for most infants during their early stages of life. Thus, if an infant is being treated within a Neonatal Intensive Care Unit (NICU), efforts are made to support and encourage new mothers in breastfeeding. However, when this is not possible hospitals will turn to alternative sources of nutrition, in most cases, donor milk provided by human milk banks (HMBs) is preferred (Hartmann et al., 2007). The amount of donor breast milk prescribed in NICUs is steadily growing (K. Carroll, 2014).

### 1.3 Bereaved mothers and their breast milk

Often, mothers will continue to lactate following the death of an infant, and so are forced to decide what to do with this milk. There are options, as reported by Dickens (2020); lactation suppression (pharmacological/nonpharmacological), lactation reduction, expression of breast milk for personal reasons and expression of breast milk with a view to donate. Breast milk has been known to hold much value and meaning for mothers and especially those whose baby has died (Fernández‐Medina et al., 2022). Welborn (2012) conducted a study investigating the experience of mothers who donated their breast milk following perinatal loss in Australia. This study found that women could fulfil some of their instincts as mothers by expressing and donating, which in turn aided the healing process. Mothers described how their lactation was a visible recognition of their move into motherhood and the act of donating gave them a tool to contemplate and process the loss of their child (Welborn, 2012).

The choice to donate breast milk is variable and personal to each mother. It has been reported that both lactation and milk donation after the loss of a baby can be seen as a way to acknowledge motherhood, and to memorialise the infant as the breast milk is directly associated with the life of the child (Cole, 2012; Layne, 2014). In Welborn's (2012) study it was noted that for those who had nursed a sick infant in the weeks or months following birth, their breast milk became a symbol of hope and survival when the infants were alive but if they had not survived, lactation was a painful reminder of the child they lost. For those whose infant had died in utero or at birth, expressing milk was a way of transitioning and maintaining a connection with their loss.

### 1.4 Rationale

Maternal grief has been shown to produce many difficult emotions (Erlandsson et al., 2011). While there are many ways grieving can be assisted, that is, having strong social support, and finding positive coping strategies, this study aims to explore mother's experience of expressing and donating breast milk within the process of grief. Considering the vast difference in breastfeeding culture between Ireland and the countries in which similar studies have been conducted it was deemed important to gain this perspective as a means of understanding the novel benefits here so that culture‐specific advice and guidance could be provided that might encourage this practice in the future.

## 2 METHODOLOGY

### 2.1 IPA

To deepen our understanding of donation behaviour within the bereaved population it is important to produce rich, descriptive data. For this, a qualitative methodology is best suited (Kint, 2015). Given the complex and sensitive nature of the subject area and the necessity of examining the participants’ meaning‐making processes, IPA was deemed to be the most appropriate method of qualitative analysis.

The process of IPA is a blend of two kinds of analyses, often described as a ‘double hermeneutic’, interpretative and phenomenological (Smith, 2004). Interpretative analyses are informed by hermeneutics; this analysis method requires the researcher to make sense and contextualise the information gathered from the participants in a way that is psychologically minded and theoretically informed. Based on the philosophical teachings of Heidegger and Gadamer, phenomenological analysis is a focus on the experience as a phenomenon, this method of analysis requires the researcher to hear and understand the lived experience of the participants’ while looking out for themes and variances to give voice to these. The melding of these two methods allows researchers to explore personal experiences in a systematic way with an idiographic focus (Smith et al., 2009).

This project lends itself particularly well to this idiographic approach. It was deemed of paramount importance to capture both likeness and divergence in the participants’ experience because one person's experience of grief is unlike anyone else's. This method allowed for these differences to be captured and valued. The COREQ checklist was used to structure this report to ensure that data was reported explicitly and comprehensively.

### 2.2 Participants

Seven individuals were recruited for this study, within the recommended 6–10 as per Smith et al. (2009). The smaller number of participants allowed the researcher to spend more time understanding each mother's meaning‐making process in detail. Creswell (2013) highlights that IPA has a focus on describing the meaning of the phenomenon in great depth and it is this which takes precedent over gaining countless points of view.

Inclusion criteria for this project were simply any mother who had chosen to donate their breast milk to the milk bank in Northern Ireland, following the bereavement of their infant. These women were recruited with support from the HMB and were all resident in Ireland or Northern Ireland when the research took place. All participants were of White ethnic background, aged between 25 and 40, co‐habiting with a partner and educated to a higher qualification level.

For four women, this loss was their first born, for two it was their second‐born child and for one mother it was her third‐born child. Three mothers experienced their child being resident in NICU before sadly passing away, and for four mothers they experienced an intrauterine death (or stillbirth). This meant that some of the women experienced expressing milk for their own children before their bereavement.

### 2.3 Procedure

#### 2.3.1 Materials

A semistructured interview schedule was used, interviews were carried out on Zoom as a means of minimising risk given the impact of the COVID‐19 pandemic. The conversations were recorded on an encrypted Dictaphone, files password protected and stored on a computer which the researcher had sole access. The audio files were transcribed onto password‐protected Microsoft Word documents; pseudonyms were then utilised to further to protect the participant's identity.

#### 2.3.2 Recruitment

This project was approved by the Office for Research Ethics Committees Northern Ireland (ORECNI). Upon donating breast milk, women are asked by the HMB to complete screening forms. Of the bereaved mothers who accessed the service since March 2020, and who indicated on the screening form that they were interested in participating in scientific research, all were contacted by telephone by a staff member from the HMB. This phone call consisted of a brief description of the study, invitation to participate in the research, and opportunity to consent to information packs being sent out in the post. Women were given contact details of the researcher (telephone and email) in the information pack and asked to contact the team if they chose to participate.

Women were given 2 weeks to make contact; if contact had not been made, a member of the milk bank staff team provided a follow‐up telephone call where potential participants could opt in or out of the study. If the individual then consented to their participation, contact details were passed onto the researcher. From here, an interview time convenient to the mother was arranged.

#### 2.3.3 Interviews

Interviews took place between June and September 2021. The interview schedule involved questions on the expression of milk, the role of the milk bank, staff support, relationship to the breast milk and decision‐making. Participants were prompted to provide a detailed narration of their experience and encouraged to share their meaning‐making process. Every discovery at interview stage is a function of the relationship between participants and the researcher, thus, time spent eliciting compassion and sincerity was a key element of this method (DeJonckheere & Vaughn, 2019).

Much attention was paid to the participants’ emotional states throughout the interviews. If a participant indicated in any way that they were feeling distressed the interview was paused and distress protocol put in place which prompted the researcher to decide whether it was appropriate to continue with the interview. Additionally, a follow‐up phone call one week after the interview was offered by the researcher as a means of checking in with those who had taken part and providing support/guidance if necessary. This was optional for participants.

#### 2.3.4 Data analysis

The process of analysing a dataset under IPA's instruction is characterised by a focus on understanding the person's lived experience. This outline was used to guide interpretation but ultimately, the author followed the research in the direction the participants initiated.

The first step required the first transcribed interview to be read and reread (Smith et al., 2009). Analytic diaries were employed by the lead researcher throughout this stage of analysis to keep note of early reflections, ideas and preconceptions. Step 2 comprised the initial annotation of the transcript, where the researcher made exploratory comments based on the transcripts. These comments comprised of subjective, contextual and linguistic information. The third step was the development of emergent themes; a means of reducing this new, much larger dataset (the interview transcript, analytic diary reflections and interview annotations) into a map of themes which represented interrelationships, connections and patterns in the data.

Step 4 involved searching for connections across the emergent themes produced in the previous step; simply an attempt to see how the themes fit together within the context of the data set. This process was repeated in that order for each interview transcript separately. Upon finishing all transcribed interviews, the final stage required the researcher to look for similar patterns across cases. Data collection, transcription, analysis and write‐up were carried out by the lead researcher alone. It is worth noting that in IPA there have been recent changes made regarding use of language and terms, considering as this project was conceptualised before these changes coming into play it was deemed appropriate to utilise the original recommendations.

#### 2.3.5 Researcher reflexivity

Reflexivity within qualitative research communicates the sensitivity with which data are collected by the researcher and in what way the research process might shape how this is interpreted to include personal experience and prior assumptions. The lead researcher in this project was an ‘outsider’ having not experienced infant loss personally. Three members of the research team identified as women and as having witnessed vicariously the devastating effects of child loss, so it could be assumed that the researchers held a particularly nurturing stance towards the participants. In terms of ontological assumptions, it is important to remember that reality is subjective and that the interpretations of the data here will have been inadvertently influenced by the researcher's perspective.

## 3 RESULTS

The rich qualitative data provided by participants were analysed. Three superordinate themes were identified: (1) Fulfilling the mother role; (2) the power of being able to ‘Do’; and (3) turning the bad into the good. These are presented separately below, see Table 1.

**Table 1:** Overall themes/subthemes identified from the analysis

	Shelly^a^	Joan	Emma	Sarah^a^	Beth^a^	Danielle	Annie^a^
1. *Fulfilling the mother role*							
‐ The need to provide for a child	X	X	X	X		X	X
‐ Expression as something only a mother can do	X	X	X	X	X	X	X
‐ The selfless self	X	X	X	X	X	X	X
‐ Breast milk as connection	X	X		X	X	X	X
2. *The power of being able to ‘DO* **’**							
‐ To be of purpose	X	X	X	X	X	X	X
‐ Altruistic behaviour (as secondary)	X	X	X	X	X	X	X
‐ Taking back control	X	X	X	X	X	X	X
3. *Making good from the bad*							
‐ Making space for the self	X	X	X	X	X	X	X
‐ Finding the positive	X	X	X			X	X

^a^ These mothers stopped expressing following the death of their infant.

### 3.1 Fulfilling the mother role

Four subthemes were identified in relation to fulfilling the mother role.

#### 3.1.1 The need to provide for a child

For the mothers in this study, the need to provide for their child was of the highest importance and related to satisfaction in motherhood. For some, expressing and providing breast milk to their child in NICU became the focus after birth.

*…well I suppose at times when (child's name) was alive it was very much focused on trying to provide his nutrition…* (Joan)

Here, Joan talked about how providing her son's nutrition was of highest importance. In this point, the mother is illustrating her wish to provide for her baby and in being able to supply this basic need she is giving her child the best chance at survival.

Emma highlighted the loss of her imagined future. This was Emma's second child and having successfully breastfed her first she had hoped to experience this again. Emma remembers the difficult parts of parenting, and how she used to find this tedious, yet in being denied this now, she longs for these moments.

*I think a huge part of it [crying], you know I should have been feeding him. I often think that you know just, I just think you know I should be feeding my baby round the clock, I should be complaining about, about cluster feeding, and that should be my life right now and it's just so cruel that, that that's been taken away from me [participant visibly upset]*. (Emma)

Dani highlighted the unique bonding experience that breastfeeding your child brings and how the lack of this added to the loss that she was faced with in the absence of her child. As another experienced mother, Dani recalled the pleasure she felt breastfeeding her previous child and so this loss felt even more pertinent.

*…and that was the one thing I was thinking all along was, ‘Aw I'll miss that bond. And I'll miss the feeding’*. (Dani)

#### 3.1.2 Expression as something only a mother can do

The ability to grow a life and give birth is something that Emma described as being of huge significance. Here, Emma talked about the wonder of the natural world and how she felt empowered in her own body's ability to grow a baby.

*…it is empowering to know that your body is, you know, giving life and is… has not just grown this life but is sustaining this little life as well in the world*. (Emma)

Annie's little girl was in a hospital incubator from birth and her care was undertaken by the staff team in NICU. Providing breast milk was the only way that Annie was able to care for her daughter, with staff having responsibility for all other needs. She referred to her breast milk having been made specifically for her daughter and how as a mother it was simply her job to ensure that her child got what she deserved.

*And it kind of, was a little pocket of time where I felt like I was doing something for her, I was able to… not… well in a way care for her by like providing something for her that nobody else could like, this was meant for her and I was able to give her that…* (Annie)

Annie went on to highlight the pleasure that she felt in fulfilling her mother role, pleasure being an important factor in how we behave it appears that Annie identified where she could receive this pleasurable feedback and placed importance on fulfilling this daily.

*Yea and you, you didn't get to do very many motherly things. You know, so it was really nice to be able to feel that*. (Annie)

Dani lost her infant very suddenly, here she discussed the physical sensation of expression and how she used this to recreate the feeling of feeding her child. For Dani, this was a significant and conscious decision.

*… I think I could feel whenever my milk was coming in, and whenever it was coming out and like, into the bottles and stuff like that, I could feel that whole process that, I suppose if a baby was feeding, em, it's the same feeling…* (Dani)

#### 3.1.3 The selfless self

The notion of a mother can denote images of an ‘all‐giving’, selfless being. The maternal instinct is one that puts a child as priority, with mum and her needs coming in second. Within this group of mothers, they utilised the provision of their breast milk to show their strength, a way to provide for their child despite hardship.

Shelly described here how she sacrificed her sleep and her energy to express milk for her baby. She referred to the immense stress that she experienced after a problematic delivery and receiving a poor prognosis for her child. Her acknowledgement of how demanding this was demonstrated the extremities to which mothers will go to when it comes to caring for their child. While recognising its difficulty, Shelly did not portray any regret in how she behaved despite the impact it had on her psychological well‐being at the time.

*…there was a lot of times when you just wanted to give up because it is so hard. Especially that, like, I was exhausted with all the stress of everything I was going through and to hear my alarm going off in the middle of the night to get up to sit and pump in the dark…* (Shelly)

For all the mothers, they spoke about the process of expressing as being very separate from the process of donating. Sarah indicated here why this might be, the maternal instinct told her she should express as a way of meeting her son's needs. She talked about the time when he was alive as being completely centered on his care and that expression played a big part in this. Donation in this case was simply a byproduct.

*…it was harder to stop expressing than what it was donating because the donations weren't for (child's name) and everything we done was based, do you know, around him emotionally*. (Sarah)

#### 3.1.4 Breast milk as connection to the child

For all the women, their breast milk represented a relationship with their child. The presence of breast milk was in some cases linked to hope, in others it represented a physical connection to their child.

Sarah described how her memory of expressing breast milk was closely linked to the memories she had of her son being alive. In a way, her breast milk represented his presence and his life. Thus, it had positive connotations for her. In her eyes, breast milk was a sign of life.

*But yea expressing was linked with him being alive and being here, so it was*. (Sarah)

For Shelly, she used the expression of her milk to hold hope that her child would become well, to stop expressing would be to give up hope. She also referred to the need to be strong for her child, as a mother she felt it was her job to be a role model for her children and in this case, remain strong so that her daughter might find the strength she needed to get better.

*…because I felt that if I stopped pumping that I was giving up hope on (child's name) like, so that's why. Part of it was just me being strong for her, not giving up, so I kept going …* (Shelly)

Annie talks about how her breast milk allowed her to build a relationship with her child, even when they were apart.

*…it kind of meant that we could spend a bit of time together in a way. Even whenever I was at home, like I would have had pictures of her, or I would have had her blanket*. (Annie)

Many of the respondents described an intense response to the physical absence of their child, whether that was because their baby was resident in NICU or because their baby had sadly passed away. For these mothers expressing gave them a sense of closeness that they otherwise would not have had. Many of the mothers used physical representations of their children such as pictures to stimulate their milk production.

The physical presence of Emma's breast milk represented the physical existence of her baby. Some of the mothers reported being advised to suppress their breast milk after their loss as a protective measure. In this group, all the women independently made the decision to express and donate and many described it as an unconscious urge that they could not simply put into words. Emma described an unshakeable emotional connection with her child and her expression of milk provided a physical connection that, in experiencing the stillbirth of her child, she would not otherwise have had opportunity to fulfil.

*I think it also allowed me to feel em… like there was still a physical connection with, with (child's name). Em, and that's why I was so reluctant to let the milk go and to follow the advice to suppress it because I thought, this is my last em, you know physical connection, like obviously he is always with us and em, I didn't feel like I needed to do it to feel close to him but I did feel like part of it was, you know it just gave me that link to my baby*. (Emma)

Three of the seven mothers continued to express after their child's death. For these women, the continuation of expressing prolonged their child's presence in their lives. They used this time to process the loss and spend time honouring their children, time they otherwise would not have had. For this reason, to stop expressing felt like another loss. Joan described how the option to donate her breast milk afforded her with more time and allowed her to viscerally experience her feelings. Joan's account also indicated that the HMB's need for breast milk was an important factor in her decision to express.

*I would have said it at the time, it was kinda… in my head my last kind of physical connection to him… [] … and knowing that I was going to help other babies that were sick, or that needed it you know. It was cathartic*. (Joan)

### 3.2 The power of being able to ‘Do’

This theme illustrated the most consistency across all respondents, three subthemes were identified.

#### 3.2.1 To be of purpose

The need to find purpose in our lives is intrinsic to our emotional well‐being. We feel good when we achieve, and demanding work brings with it satisfaction (Schaefer et al., 2013). For these mothers, the maternity leave they had imagined was sadly replaced with either immediate mourning or stress‐filled days spent in hospital. Expression was used as a way to structure these days, bringing with it containment as well as providing a space to feel of importance.

The helplessness that is faced by a mother when an infant is admitted to hospital can be overwhelming, these mothers described how they felt they had a lack of power to create change. By producing breast milk used to nourish her sick child, Sarah was able to create a positive dialogue with herself. She described here a sense of achievement in doing this.

*It felt like I was doing something good and it gave me a wee boost to, every time you seen his milk going up and stuff you knew that ‘Oh I done that’. You know? ‘I was able to do that’. So, it did, it made you feel like you were able to do something because you were so helpless in every other sense*. (Sarah)

The inability to provide basic care was a consistent thread throughout all the mothers’ accounts as being a source of frustration and disappointment. Beth described how her capacity to express milk and thus be of purpose to her daughter helped her get through this challenging time period.

*It really helped. It helps you with pushing forward, especially I suppose, because it's neonatal and things, it gives you a purpose in some respects, it gives you a boost. It makes you feel like you're actually contributing… So, in that respect… it's invaluable*. (Beth)

Annie's remark highlighted the very stark reality of having a sick baby. To feel helpless is to feel vulnerable and without the ability to create change. For her, expressing milk gave her a power that she would have otherwise not had. Annie also acknowledged how unprepared she was for this reaction which emphasizes the intensity of the situation.

*I think it just made me feel useful, instead of so helpless. I think I underestimated the power of helplessness that you feel*. (Annie)

Emma's words describe the power of her grief, she recalled how she used her expression to get out of this headspace even if only for short periods of time. Her account tells us that she understood her own needs and how it was necessary for her to have a focus outside of her grief. For Emma, the responsibility to pump was what she needed to keep going.

*I think when you're grieving and when you're in the midst of that, you need something to get you out of bed in the morning … [] … like a purpose, or em, just something to focus on where, however many times a day you have to go and express or pump em… that's your focus, and em, that… you know it just means that you're not curled up in a ball for those… for that half hour when you're pumping*. (Emma)

#### 3.2.2 Altruistic behavior (as secondary)

Donation behavior is very intricately linked to the idea of altruism. As is clear from the accounts so far, donation was not the motivation for most of the mothers in this project. It did however play a role in decision‐making.

Shelly drew attention to her concern for others, and how her ability to be of help permitted her to feel good. Shelly also displayed positive perspective taking here, something that was present throughout many of the mothers’ stories. When experiencing hardship it can help for us to see ourselves as fortunate, Shelly used accounts of other mothers’ misfortune to help her come to terms with her own situation.

*Yea, it was helpful that I was doing something for someone else, you know, and emm… and I like, a lot of the mum's have problems with breastmilk and so you know, so I was lucky that it did come in…* (Shelly)

Beth expressed milk with the sole intention of feeding her child and ensuring a good supply was established, highlighting again the maternal need to provide. The milk that was not for her baby lost its significance; donation was used simply to avoid waste. The ability to separate this could be seen as protective in the sense that it was easier to part with milk that has no sentimental value, whereas if an attachment was present, it may have felt like another loss.

*…my head was always for (child's name) to be honest. It was more, ‘I want to get it established for her, I want to keep going for her, I want it there if she needs it’. So, as much as she had some of it, the rest was just superfluous. It was just the extra that we didn't need. It didn't matter. What mattered was the fact that I had it for her*. (Beth)

Dani highlighted the influence of others; for some of the women, it elicited pleasure to share their donation story and was a way of receiving positive feedback from others. Dani echoes Beth's point in that while it was great that she could donate and help others, this was not her primary focus.

*I know people do say about the babies that you're helping but that's definitely not… of course it is whenever somebody says to you, ‘You're going to be helping babies’, but it's not at the front… at the front of your mind*. (Dani)

Sarah's very honest statement here depicts that donating to help other babies was not reason enough to express. What we have learnt is that for all our respondents, their sole focus was either to provide for their child or to provide themselves with space to process what had happened. While genuine altruism was evident in all accounts, it was always secondary to something of higher importance.

*…if you didn't have a child and I could express now, if I wanted to help other babies, would I do it? Realistically no*. (Sarah)

#### 3.2.3 Taking back control

As previously mentioned, all the women in this study felt a sense of powerlessness and lack of control at having been thrust into their situations. Anxiety is often born in the absence of control (Mandler & Watson, 1966) and thus, it is a natural response to seek control as a way of mitigating anxiety.

Shelly painted a vivid picture, conjuring images of a desperate mother by a child's bedside longing to provide aid but being unable to do so. What Shelly and all the women in this project have done is given themselves a method with which to assist and thus exert some control over the situation.

*…like when your baby's in ICU you feel completely helpless it's literally, you're just sitting there looking at them you know there's nothing you can do to help them so that's why I found the breastmilk, the breast pumping was the one little thing I could actually do…* (Shelly)

We get a sense that the women felt as though they had little control over their lives at these points, they were searching for something they could manipulate. Their ability to express breast milk became the vessel through which they could do this which helped them manage their otherwise chaotic day‐to‐day, as Joan highlighted, a choice in a world that felt choiceless.

*As I say, when you're in these situations you don't really have much of an option… much of a choice, but I suppose that [expression] was a choice*. (Joan)

Annie, a first‐time mother was shaken by an early delivery and a premature entrance into the world of parenthood. Her experience was shaped by a knowledgeable staff member who gave her options when she felt as though she had none. Throughout the mothers’ accounts, the input from staff has varied greatly. Annie was fortunate that she was guided in a direction that was of benefit to her. She highlighted the impact of safety in relationships as she was supported to try something new and scary. A positive account which highlighted the pivotal role of staff on mothers’ experiences.

*So then that actually started me, with the help of that nurse I kind of felt, kind of safe enough to start it. Because she said, ‘Look, I know you feel like you can't do anything’, ‘cos I had said that to her. ‘Em but this is something you can do’, and I thought, ‘Right, if I can do something then at least it's better than nothing’. So then I started then expressing, that's kind of how it all started*. (Annie)

Sarah's words conjure images of someone who has been robbed, robbed of motherhood and of the life that she had planned. Expressing breast milk helped her feel, if only for a short time, that she was able to take back what had been taken from her.

*Whereas if it had have been bottle fed, do you know, that's something else that the control would have been took away from you*. (Sarah)

### 3.3 Making good from the bad

#### 3.3.1 Making space for the self

The mothers’ illustrated the importance of creating space for their reactions to their individual experience. For some this involved simply telling their story, for others it was time to themselves. For all, they acknowledged the value in honouring what they needed at different time points in their journey.

The act of expressing was associated with time alone. In Beth's account, this time gave her space to be still and step away from the busyness of the NICU. Beth highlighted how important this space was as a way of calming her nervous system to continually show up for her daughter.

*…that's the kind of time when you got, you got a bit of space to yourself…* (Beth)

Joan acknowledged her need to escape her own reality. Her present life was full of tribulations, and she felt the need to remove herself from this. Throughout the family's time spent in hospital, expression was Joan's escape. Joan spoke a lot in her interview about how she utilised distraction while pumping, leaning out, when possible, of the emotion surrounding her current experience.

*…it kind of became my, I suppose my escape where I could get an escape from the hospital and still be doing something positive and giving myself a chance to wind down a bit*. (Joan)

Below, Joan illustrates in her decision to continue expressing after her son passed away, that she has created a connection to expressing. She used expression as an emotional crutch throughout her son's short life and as a successful coping skill she brought this with her into grief. Although the experience of expression after her son's death was different and for Joan a confusing mix of emotions, it still provided comfort.

*…and then when he died it was… [pause] definitely the first week I found it very… I found it hard to have to go pump. Em, at the same time it was, it still was kind of an escape but it was an escape… I was still focusing on… it was a big glaring reminder that (child's name) wasn't around anymore and it wasn't for him anymore so it was weird. It was kind of a strange mixture of feelings I suppose*. (Joan)

Emma explained her need to consciously take control of her grief. She positively used expression as an aid following the death of her child. This also permits us to ponder if there is a degree of self‐awareness necessary for this act to be of benefit.

*I'm the kind of person who, I'm not going to run away from my grief and I really need to do these different things, and talking about it, and being very kind of active in processing it. So it [expressing breastmilk] was a way of actively processing my grief*. (Emma)

On reflection, Annie spent time thinking about her experience of expression and how this helped her to grieve her daughter. She exhibited a satisfaction in her decision and acknowledged the benefits she experienced. It appears that having fulfilled her own wishes allowed her to process what had happened with somewhat more ease.

*… I think it's helped me to, to grieve, to process the grief now knowing that I did it and how I, how I felt*. (Annie)

#### 3.3.2 Finding the positive

Like perspective taking as aforementioned, finding the positive in demanding situations is a well‐known coping strategy. For most of our mothers here, they explained how they used their production of milk and its subsequent donation as a positive take on what was otherwise a tragic situation.

Shelly touched on the immense effect that her child's death had on her. She referenced her heartbreak and specifically how this was still ever present. Being able to donate her breast milk was a small thing to do in the grand scheme of her grief but something she simply had to do.

*…because I was absolutely heartbroken you know, and still am, you know it's… But as I said like, I had all the milk there like I had to like, I couldn't… I didn't have the heart to you know… I had to do something with it*. (Shelly)

Joan depicted a need to find a positive in her situation. She communicated that this was a constant thought in her mind and that she felt a pressure to channel her energy into something constructive. It appeared as though Joan had been proactive on her journey and she demonstrated an optimistic mindset.

*I kept saying ‘I have to find something positive in all of this’*. (Joan)

By expressing her breast milk Emma created positivity in what was otherwise a desperate situation. A constant presence interwoven through the interviews was the wish to seek lightness. It seemed that even in the most traumatic of times these women recognised their ability to create something good, instilling further their autonomy and power to create change.

*… and it just like I said brings positivity into a really traumatic situation*. (Emma)

Dani was one mother who enjoyed the physical act of expressing, she shared this experience with her family and as a unit they used it to grieve the loss of the infant. For Dani, expression brought positivity to her days. Her analogy depicts a very real image of a mother overridden with grief. What she tells us here is not that this was never present, but that during expression she was freed from this grief for a finite amount of time.

*And I was kind of conscious that if I did, if it was making me upset every time I was pumping, if I was filling the bottles with tears more than milk, then that was never going to be a good thing for me, em… for my mental health. But I knew that… I didn't get to that stage ever thankfully it was just a… I enjoyed doing it…* (Dani)

## 4 DISCUSSION

### 4.1 Summary

An American novelist once wrote; ‘sometimes the strength of motherhood is greater than natural laws’ (Kingsolver, 2020). This study summarises the accounts of seven women who were faced with unimaginable pain in grief. Through their stories, they shared their intimate experiences of motherhood during a time of suffering, and how they used being a mother as a mechanism to carry on. When faced with grief, they used their ability to provide through expression as a way to cope. The power of being able to do something gave them a sense of control in a world where they didn*'*t have any. Expression created structure, and routine which afforded them with a feeling of autonomy and containment. For the majority of the women, the wish to create something positive from their experience motivated them to keep going through difficult days and nights, it gave them space to foster hope for the future and time to process the magnitude of what was going on around them.

### 4.2 Interpretations

‘To be a mother’ was of utmost importance to the women in this project, they exhibited a visceral urge to fulfil the varying roles associated with motherhood. The ‘motherhood myth’ is a concept that has been examined in recent years, a societal pressure which poses that mothers’ are solely responsible for the development and wellbeing of their children, often leading to maternal guilt when mothers don't meet this high standard (Constantinou et al., 2021). There are many ways in which a mother might fulfil this role, for lots of women this can be in the form of providing food, warmth, or physical affection; for the mothers here, their provision was encapsulated in their breast milk. With breast milk being the most natural and age‐old form of provision from mother to child, this act feels instinctive. While much breastfeeding literature focuses on the physiological benefits of breastfeeding, it is more recently acknowledged that this also satisfies social and psychological nourishment for both mothers and babies (Dykes & Flacking, 2010), creating that ‘feel good’ and making space for attachment bonds to form.

The gift of producing breast milk is parallel to that of the ability to bear children. Within the Irish culture when a woman becomes pregnant, the excitement and anticipation is focused on the manifestation of the unborn child (Gallegos et al., 2020), with little emphasis placed on lactation and its upcoming role. For these women, when they did not meet their imagined future of being with their child, breast milk stepped in to fill this space. Expression appeared to go some way in bridging the gap between expectant and realised motherhood.

Finding purpose in life predicts both longevity and good health (Steptoe et al., 2015). The ability to find meaning, most particularly when faced with challenges is an important mechanism for resilience, and thus finding purpose can be a significant predictor for better emotional recovery following trauma (Schaefer et al., 2013). Here, expression brought purpose, meaning and structure. An essential element of this was in the sense of control that these women gained. Correlations between control and anxiety are well‐known, where actual or appraised lack of control leads to increased anxiety (Mandler & Watson, 1966; Watson, 1967). Taking control by expressing and donating their breast milk consequently gave these women ownership, and reduced their experience of anxiety.

This research was exploratory in the sense that it hoped to gain an understanding of how mothers used their donating behaviour in grief. Our expectations of this research were focused on that of understanding how women experienced perinatal loss and how they sought comfort. Previous research by Welborn (2012) highlighted that bereaved mothers’ exhibit a need to nurture. Our research compliments this as well as providing an interesting paradox; if someone carried out an act of altruism and later explained that this was to benefit themselves, could it really be described as altruistic?

Prosocial behaviour, or altruism, refers to acting for the benefit of another with a cost to the individual fulfilling the act of giving (Diacon, 2014). Each of the women in this study discussed the act of donating as important and that it brought with it a sense of achievement and pride. In their accounts, while the idea of helping other mothers and sick babies was used as a motivator, these women were clear to differentiate that this was secondary to their own personal gain.

Described by one participant as ‘unapologetic selfishness’, the idea of making choices that essentially served themselves was a regular theme through the interviews and could be easily reframed as self‐compassion. Research by Vara and Thimm (2020) found that low rates of self‐compassion in bereaved individuals led to an increased severity of complicated grief symptoms. This led us to believe that by honouring their own needs, these women helped to reduce the psychological distress of their grief, even if only for the short time period that they expressed.

Research tells us that trust is possible only in an effective therapeutic relationship (Fonagy & Allison, 2014). The women in this study exhibited great openness in their accounts which told us that they felt safe, credit can be given here to the researcher–participant relationship. Utilising the interview as a space to tell their story was pertinent in all the mother's accounts. In line with Kauffman's (2013) teachings that the process of grieving involves storytelling, reminiscing and recreating meaning, these women engaged in this simply through participating in this project.

### 4.3 Limitations

The women in this study were homogenous in nature, limiting transferability; all being White women aged 25–40 and residing in Ireland or Northern Ireland. While this could be seen as a limitation, it is important to note that this is representative of the breastfeeding population within Ireland. The virtual method of data collection may also be recognised as a limitation here.

Each of these mothers were supported by their partners, extended family, friends and occasionally accommodating midwifery staff. This care afforded them with the luxury of time, and positive affirmations surrounding their decision to express their breast milk. With many child‐bearing women in differing personal circumstances and with ever‐present discrepancies within the health care systems, we cannot assume that all women will be supported in this way. The sensitive nature of perinatal loss requires much care and consideration, and we advise exercising caution on encouraging mothers to do something that may feel unnatural to them especially if the necessary supports are not in place.

Another factor to consider was the presence of COVID‐19; all the mothers experienced the effects of COVID‐19 on their journeys through the care systems. Their experience of both perinatal sickness and grief was shaped by COVID‐19 and its restrictions. In terms of the research itself, all interviews were conducted virtually which meant that the researcher needed to exercise increased caution with the interview style. The inability to be physically present with the participants meant that more attention needed to be paid to the well‐being of these mothers. Despite this, the distress protocol did not need to be utilised with the women showing incredible strength and resilience throughout their interviews.

### 4.4 Recommendations

This study highlighted the increased need for informed decision‐making regarding lactation after perinatal loss. It is recommended that when a mother experiences a perinatal loss she is given all the relevant information regarding her production of milk and the options available to her. This research points out that for these women, expression served as a powerful tool in helping them process the sickness/death of their child and so it can be assumed that this could be beneficial to many more women in the future.

This responsibility lies with health care staff, and so increased awareness of the possible benefits of expression must be disseminated across staff teams to ensure professionals are armed with the knowledge they need to support mothers appropriately. An essential element of this is the need for more open, compassionate, and sensitive conversations between staff and mothers. It is not an arena for judgement, only kindness and support. Many staff members report feeling unequipped to have these conversations, however contrary to belief they require no great skill only compassion and human understanding. Ultimately, the decision to express/donate is individual to each woman, and this should be respected above all. By providing a nonpersuasive, informative and safe space, we can enable women to make a decision that is right for them.

This research project is the first of its kind to take place in the United Kingdom/Ireland. The analysis hinted that there may be differences in the experience of expressing breast milk to nurture a sick child versus expressing following the death of a child. Additional research exploring this would be beneficial and further our understanding of the subject area.

## 5 CONCLUSION

This study was important in highlighting the increased need to recognise lactation following perinatal loss as a significant factor in a mother's grief. Expression of breast milk played a powerful and positive role for all the participants in this study. Mothers require support from health care staff to be armoured with the necessary information to make informed decisions following perinatal loss. For the women in this project, the expression and donation of breast milk was a healing ritual that allowed them to at least partially fulfil their mothering role, be of importance and create positive associations with their loss. The time and energy spent honouring their infant in this way was essential in the acceptance and sense‐making process following their bereavement and allowed for the integration of this loss into their lives and new identity.

## AUTHOR CONTRIBUTIONS

Gráinne Ward and Nicola Doherty conceived the idea presented in this project. Gráinne Ward developed the protocol, conducted the data collection and performed the analysis. Pauline Adair verified the analytical methods and supervised the findings of this work. Nicola Doherty supervised the clinical reflections pertinent in this project. All authors contributed to the final manuscript by providing guidance and feedback.

## CONFLICTS OF INTEREST

There are no conflicts of interest in this project. The study was completed as part of a clinical doctorate psychology programme and the research was undertaken in order to improve the field of research in perinatal psychology. The research team were not paid to complete this project outside of their salaries.

## Data Availability

The data that support the findings of this study are available on request from the corresponding author. The data are not publicly available due to privacy or ethical restrictions.
